# Supplementary material for: A synthetic biology approach to probing nucleosome symmetry
Source: eLife. 2017 Sep 12;6:e28836. doi: 10.7554/eLife.28836 (PMC5626479; doi:10.7554/eLife.28836)
Supplement: Supplementary table 1. [file elife-28836-fig1.docx]

**Strain list**

| **Strain** | **Description** | **Parent** | **Reference** |
| --- | --- | --- | --- |
| PKY4171 | MAT**a**; Δ(*hht1-hhf1*); Δ(*hht2-hhf2*); *leu2-3,112*; *ura3-62*; *trp1*; *his3*; *bar1::hisG+ p(HHT1-HHF1, URA3, CEN)* | MSY421 | (Recht *et al.* PNAS 2006) |
|  |  |  |  |
| PKY4574 | +P65 (*BirA::HIS3* integration plasmid) | PKY4171 | This study |
| PKY4605 | +pH3X (H3X = hht2(L126A, L130V), HHF2, TRP1)  + P54 (biotin-H3Y = biotin-hht2(L109I, A110W, L130I), HHF2, LEU2) | PKY4574 | This study |
| PKY4610  (WT wt-C) | + P66 (bio-H3 = biotin-hht2, HHF2, TRP1) | PKY4574 | This study |
| PKY4616 | + P66 (bio-H3 = biotin-hht2, HHF2, TRP1)  + P30 (V5-H3 = V5-hht2, HHF2, URA3) | PKY4171 | This study |
| PKY4625 | + P22 (Myc-H3Y = Myc-hht2(L109I, A110W, L130I), HHF2, LEU2)  + P67 (bio-H3X-126A = biotin-hht2(L126A, L130V), HHF2, TRP1)  + P72 (V5-H3X-126A = V5-hht2(L126A, L130V), HHF2, URA3) | PKY4171 | This study |
| PKY4694 | + P54 (biotin-H3Y = biotin-hht2(L109I, A110W, L130I), HHF2, LEU2)  + P72 (V5-H3X-126A = V5-hht2(L126A, L130V), HHF2, URA3)  + P35 (Myc-H3Y = Myc-hht2(L109I, A110W, L130I), HHF2, TRP1) | PKY4171 | This study |
| PKY4701  (WT wt-c) | MAT**a**; Δ(*hht-hhf1*); Δ(*hht2-hhf2*); *leu2-3,112*; *ura3-62*; *trp1*; *his3*;  + pPK188 (HHT2-HHF2, URA3, CEN) | PKY4130 | This study |
| PKY4704  (pWT XY) | +pH3X (H3X = hht2(L126A, L130V), HHF2, TRP1)  +pH3Y (H3Y = hht2(L109I, A110W, L130I), HHF2, LEU2) | PKY4701 | This study |
| PKY4749  (R2K mXY) | +P93 (H3X-R2K = hht2(R2K, L126A, L130V), HHF2, TRP1)  + pH3Y (H3Y = hht2(L109I, A110W, L130I), HHF2, LEU2) | PKY4701 | This study |
| PKY4751  (R2K XmY) | + pH3X (H3X = hht2(L126A, L130V), HHF2, TRP1)  +P94 (H3Y-R2K = hht2(R2K, L109I, A110W, L130I), HHF2, LEU2) | PKY4701 | This study |
| PKY4753  (R2K mXmY) | +P93 (H3X-R2K = hht2(R2K, L126A, L130V), HHF2, TRP1)  +P94 (H3Y-R2K = hht2(R2K, L109I, A110W, L130I), HHF2, LEU2) | PKY4701 | This study |
| PKY4714  (K9Q mXY) | +P81 (H3X-K9Q = hht2(K9Q, L126A, L130V), HHF2, TRP1)  + pH3Y (H3Y = hht2(L109I, A110W, L130I), HHF2, LEU2) | PKY4701 | This study |
| PKY4715  (K9Q XmY) | + pH3X (H3X = hht2(L126A, L130V), HHF2, TRP1)  +P84 (H3Y-K9Q = hht2(K9Q, L109I, A110W, L130I), HHF2, LEU2) | PKY4701 | This study |
| PKY4706  (K9Q mXmY) | +P81 (H3X-K9Q = hht2(K9Q, L126A, L130V), HHF2, TRP1)  +P84 (H3Y-K9Q = hht2(K9Q, L109I, A110W, L130I), HHF2, LEU2) | PKY4701 | This study |
| PKY4773  (K9R mXY) | +P102 (H3X-K9R = hht2(K9R, L126A, L130V), HHF2, TRP1)  + pH3Y (H3Y = hht2(L109I, A110W, L130I), HHF2, LEU2) | PKY4701 | This study |
| PKY4775  (K9R XmY) | + pH3X (H3X = hht2(L126A, L130V), HHF2, TRP1)  +P103 (H3Y-K9R = hht2(K9R, L109I, A110W, L130I), HHF2, LEU2) | PKY4701 | This study |
| PKY4777  (K9R mXmY) | +P102 (H3X-K9R = hht2(K9R, L126A, L130V), HHF2, TRP1)  +P103 (H3Y-K9R = hht2(K9R, L109I, A110W, L130I), HHF2, LEU2) | PKY4701 | This study |
| PKY4716  (S10A mXY) | +P82 (H3X-S10A = hht2(S10A, L126A, L130V), HHF2, TRP1)  + pH3Y (H3Y = hht2(L109I, A110W, L130I), HHF2, LEU2) | PKY4701 | This study |
| PKY4717  (S10A XmY) | + pH3X (H3X = hht2(L126A, L130V), HHF2, TRP1)  +P85 (H3Y-S10A = hht2(S10A, L109I, A110W, L130I), HHF2, LEU2) | PKY4701 | This study |
| PKY4707  (S10A mXmY) | +P82 (H3X-S10A = hht2(S10A, L126A, L130V), HHF2, TRP1)  +P85 (H3Y-S10A = hht2(S10A, L109I, A110W, L130I), HHF2, LEU2) | PKY4701 | This study |
| PKY4787  (K14Q wt-c) | + p107 (H3-K14Q = hht2(K14Q), HHF2, TRP1)  +pRS415 | PKY4701 | This study |
| PKY4789  (K14Q mXY) | +P108 (H3X-K14Q = hht2(K14Q, L126A, L130V), HHF2, TRP1)  + pH3Y (H3Y = hht2(L109I, A110W, L130I), HHF2, LEU2) | PKY4701 | This study |
| PKY4791  (K14Q XmY) | + pH3X (H3X = hht2(L126A, L130V), HHF2, TRP1)  +P109 (H3Y-K14Q = hht2(K14Q, L109I, A110W, L130I), HHF2, LEU2) | PKY4701 | This study |
| PKY4793  (K14Q mXmY) | +P108 (H3X-K14Q = hht2(K14Q, L126A, L130V), HHF2, TRP1)  +P109 (H3Y-K14Q = hht2(K14Q, L109I, A110W, L130I), HHF2, LEU2) | PKY4701 | This study |
| PKY4779  (K14R wt-c) | + p104 (H3-K14R = hht2(K14R), HHF2, TRP1)  +pRS415 | PKY4701 | This study |
| PKY4781  (K14R mXY) | +P105 (H3X-K14R = hht2(K14R, L126A, L130V), HHF2, TRP1)  + pH3Y (H3Y = hht2(L109I, A110W, L130I), HHF2, LEU2) | PKY4701 | This study |
| PKY4783  (K14R XmY) | + pH3X (H3X = hht2(L126A, L130V), HHF2, TRP1)  +P106 (H3Y-K14R = hht2(K14R, L109I, A110W, L130I), HHF2, LEU2) | PKY4701 | This study |
| PKY4786  (K14R mXmY) | +P105 (H3X-K14R = hht2(K14R, L126A, L130V), HHF2, TRP1)  +P106 (H3Y-K14R = hht2(K14R, L109I, A110W, L130I), HHF2, LEU2) | PKY4701 | This study |
| PKY4805  (K18Q mXY) | +P114 (H3X-K18Q = hht2(K18Q, L126A, L130V), HHF2, TRP1)  + pH3Y (H3Y = hht2(L109I, A110W, L130I), HHF2, LEU2) | PKY4701 | This study |
| PKY4807  (K18Q XmY) | + pH3X (H3X = hht2(L126A, L130V), HHF2, TRP1)  +P115 (H3Y-K18Q = hht2(K18Q, L109I, A110W, L130I), HHF2, LEU2) | PKY4701 | This study |
| PKY4809  (K18Q mXmY) | +P114 (H3X-K18Q = hht2(K18Q, L126A, L130V), HHF2, TRP1)  +P115 (H3Y-K18Q = hht2(K18Q, L109I, A110W, L130I), HHF2, LEU2) | PKY4701 | This study |
| PKY4797  (K18R mXY) | +P111 (H3X-K18R = hht2(K18R, L126A, L130V), HHF2, TRP1)  + pH3Y (H3Y = hht2(L109I, A110W, L130I), HHF2, LEU2) | PKY4701 | This study |
| PKY4799  (K18R XmY) | + pH3X (H3X = hht2(L126A, L130V), HHF2, TRP1)  +P112 (H3Y-K18R = hht2(K18R, L109I, A110W, L130I), HHF2, LEU2) | PKY4701 | This study |
| PKY4801  (K18R mXmY) | +P111 (H3X-K18R = hht2(K18R, L126A, L130V), HHF2, TRP1)  +P112 (H3Y-K18R = hht2(K18R, L109I, A110W, L130I), HHF2, LEU2) | PKY4701 | This study |
| PKY4822  (K27Q mXY) | +P120 (H3X-K27Q = hht2(K27Q, L126A, L130V), HHF2, TRP1)  + pH3Y (H3Y = hht2(L109I, A110W, L130I), HHF2, LEU2) | PKY4701 | This study |
| PKY4823  (K27Q XmY) | + pH3X (H3X = hht2(L126A, L130V), HHF2, TRP1)  +P121 (H3Y-K27Q = hht2(K27Q, L109I, A110W, L130I), HHF2, LEU2) | PKY4701 | This study |
| PKY4825  (K27Q mXmY) | +P120 (H3X-K27Q = hht2(K27Q, L126A, L130V), HHF2, TRP1)  +P121 (H3Y-K27Q = hht2(K27Q, L109I, A110W, L130I), HHF2, LEU2) | PKY4701 | This study |
| PKY4813  (K27R mXY) | +P127 (H3X-K27R = hht2(K27R, L126A, L130V), HHF2, TRP1)  + pH3Y (H3Y = hht2(L109I, A110W, L130I), HHF2, LEU2) | PKY4701 | This study |
| PKY4815  (K27R XmY) | + pH3X (H3X = hht2(L126A, L130V), HHF2, TRP1)  +P128 (H3Y-K27R = hht2(K27R, L109I, A110W, L130I), HHF2, LEU2) | PKY4701 | This study |
| PKY4817  (K27R mXmY) | +P127 (H3X-K27R = hht2(K27R, L126A, L130V), HHF2, TRP1)  +P128 (H3Y-K27R = hht2(K27R, L109I, A110W, L130I), HHF2, LEU2) | PKY4701 | This study |
| PKY4827  (K36Q wt-c) | + p122 (H3-K36Q = hht2(K36Q), HHF2, TRP1)  +pRS415 | PKY4701 | This study |
| PKY4829  (K36Q mXY) | +P123 (H3X-K36Q = hht2(K36Q, L126A, L130V), HHF2, TRP1)  + pH3Y (H3Y = hht2(L109I, A110W, L130I), HHF2, LEU2) | PKY4701 | This study |
| PKY4831  (K36Q XmY) | + pH3X (H3X = hht2(L126A, L130V), HHF2, TRP1)  +P124 (H3Y-K36Q = hht2(K36Q, L109I, A110W, L130I), HHF2, LEU2) | PKY4701 | This study |
| PKY4834  (K36Q mXmY) | +P123 (H3X-K36Q = hht2(K36Q, L126A, L130V), HHF2, TRP1)  +P124 (H3Y-K36Q = hht2(K36Q, L109I, A110W, L130I), HHF2, LEU2) | PKY4701 | This study |
| PKY4837  (K37Q mXY) | +P126 (H3X-K37Q = hht2(K37Q, L126A, L130V), HHF2, TRP1)  + pH3Y (H3Y = hht2(L109I, A110W, L130I), HHF2, LEU2) | PKY4701 | This study |
| PKY4839  (K37Q XmY) | + pH3X (H3X = hht2(L126A, L130V), HHF2, TRP1)  +P127 (H3Y-K37Q = hht2(K37Q, L109I, A110W, L130I), HHF2, LEU2) | PKY4701 | This study |
| PKY4841  (K37Q mXmY) | +P126 (H3X-K37Q = hht2(K37Q, L126A, L130V), HHF2, TRP1)  +P127 (H3Y-K37Q = hht2(K37Q, L109I, A110W, L130I), HHF2, LEU2) | PKY4701 | This study |
| PKY4843  (K56R wt-c) | + p128 (H3-K56R = hht2(K56R), HHF2, TRP1)  +pRS415 | PKY4701 | This study |
| PKY4983  (pWT-XY) | +P67 (bio-H3X = bio-hht2(L126A, L130V), HHF2, TRP1)  + pH3Y (H3Y = hht2(L109I, A110W, L130I), HHF2, LEU2) | PKY4574 | This study |
| PKY4986  (S10A XY trans) | +P67 (bio-H3X = bio-hht2(L126A, L130V), HHF2, TRP1)  + p85 (H3Y-S10A = hht2(S10A, L109I, A110W, L130I), HHF2, LEU2) | PKY4574 | This study |
| PKY5003  (S10A wt-C) | + P165 (bio-H3-S10A= biotin-hht2(S10A), HHF2, TRP1)  +pRS415 | PKY4574 | This study |
| PKY5005  (S10A XY cis) | +P166 (bio-H3X-S10A = bio-hht2(S10A, L126A, L130V), HHF2, TRP1)  + pH3Y (H3Y = hht2(L109I, A110W, L130I), HHF2, LEU2) | PKY4574 | This study |
| PKY5031  (P38V wt-c) | + p160 (H3-P38V = hht2(P38V), HHF2, TRP1)  +pRS415 | PKY4701 | This study |
| PKY5033  (P38V mXY) | +P161 (H3X-P38V = hht2(P38V, L126A, L130V), HHF2, TRP1)  + pH3Y (H3Y = hht2(L109I, A110W, L130I), HHF2, LEU2) | PKY4701 | This study |
| PKY5035  (P38V XmY) | + pH3X (H3X = hht2(L126A, L130V), HHF2, TRP1)  +P162 (H3Y- P38V = hht2(P38V, L109I, A110W, L130I), HHF2, LEU2) | PKY4701 | This study |
| PKY5037  (P38V mXmY) | +P161 (H3X-P38V = hht2(P38V, L126A, L130V), HHF2, TRP1)  +P162 (H3Y- P38V = hht2(P38V, L109I, A110W, L130I), HHF2, LEU2) | PKY4701 | This study |
| PKY5042  (S10A XY double) | +P166 (bio-H3X-S10A = bio-hht2(S10A, L126A, L130V), HHF2, TRP1)  + p85 (H3Y-S10A = hht2(S10A, L109I, A110W, L130I), HHF2, LEU2) | PKY4574 | This study |
| PKY5077  (*eaf3Δ* WT wt-c) | MAT**a**; Δ(*hht-hhf1*); Δ(*hht2-hhf2*); *leu2-3,112*; *ura3-62*; *trp1*; *his3*; *eaf3::KanMX4* + pPK188 (HHT2-HHF2, URA3, CEN), isolate 1 | PKY4701 | This study |
| PKY5078  (*eaf3Δ* WT wt-c) | MAT**a**; Δ(*hht-hhf1*); Δ(*hht2-hhf2*); *leu2-3,112*; *ura3-62*; *trp1*; *his3*; *eaf3::KanMX4* + pPK188 (HHT2-HHF2, URA3, CEN), isolate 2 | PKY4701 | This study |
| PKY5079  (*eaf3Δ* K36Q wt-c) | + p122 (H3-K36Q = hht2(K36Q), HHF2, TRP1)  +pRS415 | PKY5077 | This study |
| PKY5081  (*eaf3Δ* pWT XY) | +pH3X (H3X = hht2(L126A, L130V), HHF2, TRP1)  +pH3Y (H3Y = hht2(L109I, A110W, L130I), HHF2, LEU2) | PKY5077 | This study |
| PKY5083  (*eaf3Δ* K36Q mXY) | +P123 (H3X-K36Q = hht2(K36Q, L126A, L130V), HHF2, TRP1)  + pH3Y (H3Y = hht2(L109I, A110W, L130I), HHF2, LEU2) | PKY5077 | This study |
| PKY5086  (*eaf3Δ* K36Q XmY) | + pH3X (H3X = hht2(L126A, L130V), HHF2, TRP1)  +P124 (H3Y-K36Q = hht2(K36Q, L109I, A110W, L130I), HHF2, LEU2) | PKY5078 | This study |
| PKY5087  (*eaf3Δ* K36Q mXmY) | +P123 (H3X-K36Q = hht2(K36Q, L126A, L130V), HHF2, TRP1)  +P124 (H3Y-K36Q = hht2(K36Q, L109I, A110W, L130I), HHF2, LEU2) | PKY5077 | This study |
| PKY5138  (K36Q_X/P38V_Y) | +P123 (H3X-K36Q = hht2(K36Q, L126A, L130V), HHF2, TRP1)  +P162 (H3Y- P38V = hht2(P38V, L109I, A110W, L130I), HHF2, LEU2) | PKY4701 | This study |
| PKY5140  (P38V_X/K36Q_Y) | +P161 (H3X-P38V = hht2(P38V, L126A, L130V), HHF2, TRP1)  +P124 (H3Y-K36Q = hht2(K36Q, L109I, A110W, L130I), HHF2, LEU2) | PKY4701 | This study |
